# Cardioprotection and Heart Failure Management in Duchenne and Becker Muscular Dystrophies: A State-of-the-Art Review Across Pediatric and Transitional Age

**DOI:** 10.3390/jcm15218554

**Published:** 2026-11-03

**Authors:** Mattia Pasquinucci, Maria Elena Derchi, Alessandra Siboldi, Sara Bondanza, Chiara Panicucci, Federica Trucco, Claudio Bruno, Davide Meneghesso

**Affiliations:** 1 Department of Pediatrics, San Bassiano Hospital, AULSS 7 Pedemontana, 36061 Bassano del Grappa, Italy; 2 Department of Pediatric Cardiology, IRCCS Istituto Giannina Gaslini, 16147 Genoa, Italy; 3 Centre of Translational and Experimental Myology, IRCCS Istituto Giannina Gaslini, 16147 Genoa, Italy; 4 Pediatric Neurology and Muscular Diseases Unit, IRCCS Istituto Giannina Gaslini, 16147 Genoa, Italy; 5 Department of Neuroscience, Rehabilitation, Ophthalmology, Genetics, Maternal and Child Health—DINOGMI, University of Genoa, 16147 Genoa, Italy

**Keywords:** Duchenne muscular dystrophy, Becker muscular dystrophy, dilated cardiomyopathy, heart failure, global longitudinal strain, ACE-inhibitors, sodium-glucose transporter 2 inhibitors, sacubitril-valsartan, transitional care, children, pediatric

## Abstract

Advances in respiratory care have significantly extended the life expectancy of patients with Duchenne and Becker muscular dystrophies (DMD/BMD), making progressive dilated cardiomyopathy the leading cause of mortality in this population. Historically, a conservative “watch and wait” approach was often maintained until overt systolic dysfunction became evident. However, the advent of advanced echocardiographic technologies, particularly Global Longitudinal Strain (GLS) imaging, has demonstrated that myocardial impairment begins in early childhood, long before a decline in ejection fraction occurs. This critical evidence strongly advocates for a paradigm shift toward early, proactive cardioprotective strategies. As patients progress into the transitional age and adulthood, the pediatric prophylactic approach (traditionally based on ACE inhibitors and beta-blockers) must integrate with evolving adult heart failure guidelines. This comprehensive review critically evaluates the pathophysiological continuum of dystrophic cardiomyopathy. We highlight the prognostic value of early subclinical detection (GLS) and specifically discuss the transition to modern guideline-directed medical therapies (GDMTs), exploring the emerging role of Angiotensin Receptor-Neprilysin Inhibitors (ARNI, e.g., sacubitril/valsartan) and SGLT2 inhibitors (e.g., dapagliflozin) in this specific cohort. Finally, we address the role of advanced device therapies (ICD, CRT, and left ventricular assist devices) in end-stage disease. By bridging pediatric neurology and adult cardiology, this review aims to equip clinicians with an updated, multidisciplinary framework to optimize cardiovascular outcomes in the growing population of transitional-age DMD/BMD patients.

## 1. Introduction

Duchenne and Becker muscular dystrophies (DMD/BMD) are X-linked neuromuscular disorders caused by pathogenic variants in the *DMD* gene and represent a spectrum of dystrophin-associated disease with highly variable skeletal and cardiac phenotypes. Over recent decades, substantial advances in respiratory care and supportive management have prolonged survival, progressively changing the clinical profile of these patients. As a consequence, cardiac involvement—particularly progressive dystrophin-associated cardiomyopathy and its associated arrhythmic complications—has emerged as a major determinant of morbidity and mortality [1,2].

This epidemiological evolution has exposed an important challenge in cardiovascular care. Despite increasing recognition of cardiac involvement, surveillance and implementation of cardioprotective therapy remain heterogeneous, and treatment has historically been driven largely by the detection of overt left ventricular systolic dysfunction or symptomatic heart failure. This approach is increasingly difficult to reconcile with the natural history of dystrophin-associated cardiomyopathy. Contemporary echocardiographic and cardiovascular magnetic resonance (CMR) studies indicate that myocardial injury, regional mechanical abnormalities, and fibrotic remodeling may become detectable years before a clinically relevant decline in left ventricular ejection fraction (LVEF) [1,3,4].

The temporal dissociation between myocardial injury and conventional measures of ventricular function has important therapeutic implications. Global LVEF remains a clinically useful parameter, but it may underestimate early and regionally heterogeneous myocardial disease. Accordingly, advanced markers such as global longitudinal strain (GLS), CMR-derived strain, late gadolinium enhancement (LGE), and parametric mapping have progressively gained importance for the identification and characterization of subclinical cardiac involvement. At the same time, evidence supporting early renin–angiotensin system inhibition and mineralocorticoid receptor antagonism has strengthened the rationale for initiating cardioprotective therapy before overt systolic dysfunction develops.

A further challenge is represented by the transition from pediatric to adult cardiovascular care. As patients with DMD increasingly survive into adolescence and young adulthood, clinical management must reconcile dystrophinopathy-specific evidence generated predominantly in pediatric populations with heart failure strategies developed largely in adults with non-dystrophic cardiomyopathy. The applicability of contemporary guideline-directed medical therapy (GDMT), including angiotensin receptor–neprilysin inhibition and sodium-glucose cotransporter-2 (SGLT2) inhibition, remains incompletely defined in DMD/BMD. Similarly, the role and timing of ivabradine, implantable cardioverter-defibrillators, cardiac resynchronization therapy, and mechanical circulatory support require individualized assessment of myocardial fibrosis, ventricular function, arrhythmic risk, respiratory status, and overall disease burden [2].

Against this background, the cardiovascular management of DMD/BMD is moving from a predominantly LVEF-driven model toward an approach increasingly informed by the biological and imaging phenotype of myocardial disease. In this State-of-the-Art Review, we synthesize the current evidence across this continuum, from early subclinical myocardial injury and preventive cardioprotection to contemporary heart failure therapy and advanced device-based strategies. Particular emphasis is placed on the integration of CMR and myocardial strain assessment with pharmacological decision-making, the emerging but still limited evidence for contemporary HF therapies in dystrophinopathy, and the specific challenges encountered during the transition from pediatric to adult care. Throughout the review, we distinguish established clinical evidence from emerging and experimental strategies in order to clarify where current practice is supported by evidence and where important knowledge gaps remain.

### Literature Search Strategy

To provide a comprehensive overview of the evolving landscape of DMD/BMD cardiovascular management, a targeted literature search was conducted using the MEDLINE (PubMed) database. The search spanned from January 2000 to September 2026, capturing the modern era of advanced cardiovascular imaging and the introduction of contemporary heart failure pharmacotherapies. The search strategy utilized a combination of Medical Subject Headings (MeSH) and free-text keywords covering four main domains: the target population (e.g., “Duchenne”, “Becker”, “dystrophinopathy”), cardiovascular phenotype (“Cardiomyopathy, Dilated”, “Heart Failure”), early diagnostic modalities (“Global Longitudinal Strain”, “CMR”, “Late Gadolinium Enhancement”), and therapeutic interventions (“Angiotensin-Converting Enzyme Inhibitors”, “ARNI”, “SGLT2i”, “Defibrillators, Implantable”).

Because the available literature is heterogeneous and includes randomized clinical trials, prospective and retrospective observational studies, registry analyses, case series, case reports, preclinical studies, and mechanistic investigations, the evidence was synthesized narratively rather than according to formal systematic-review methodology. Priority was given to prospective clinical studies, randomized trials, large observational cohorts, contemporary registries, consensus documents, and recent cardiovascular magnetic resonance studies, while lower-level evidence was considered when addressing emerging or experimental therapies for which clinical data remain limited.

The aim of the search was therefore not to provide a quantitative estimate of treatment effects, but to integrate the most clinically relevant and contemporary evidence across the full spectrum of dystrophin-associated cardiac disease, from subclinical myocardial injury to advanced heart failure and transitional-age care.

**Figure 1:**
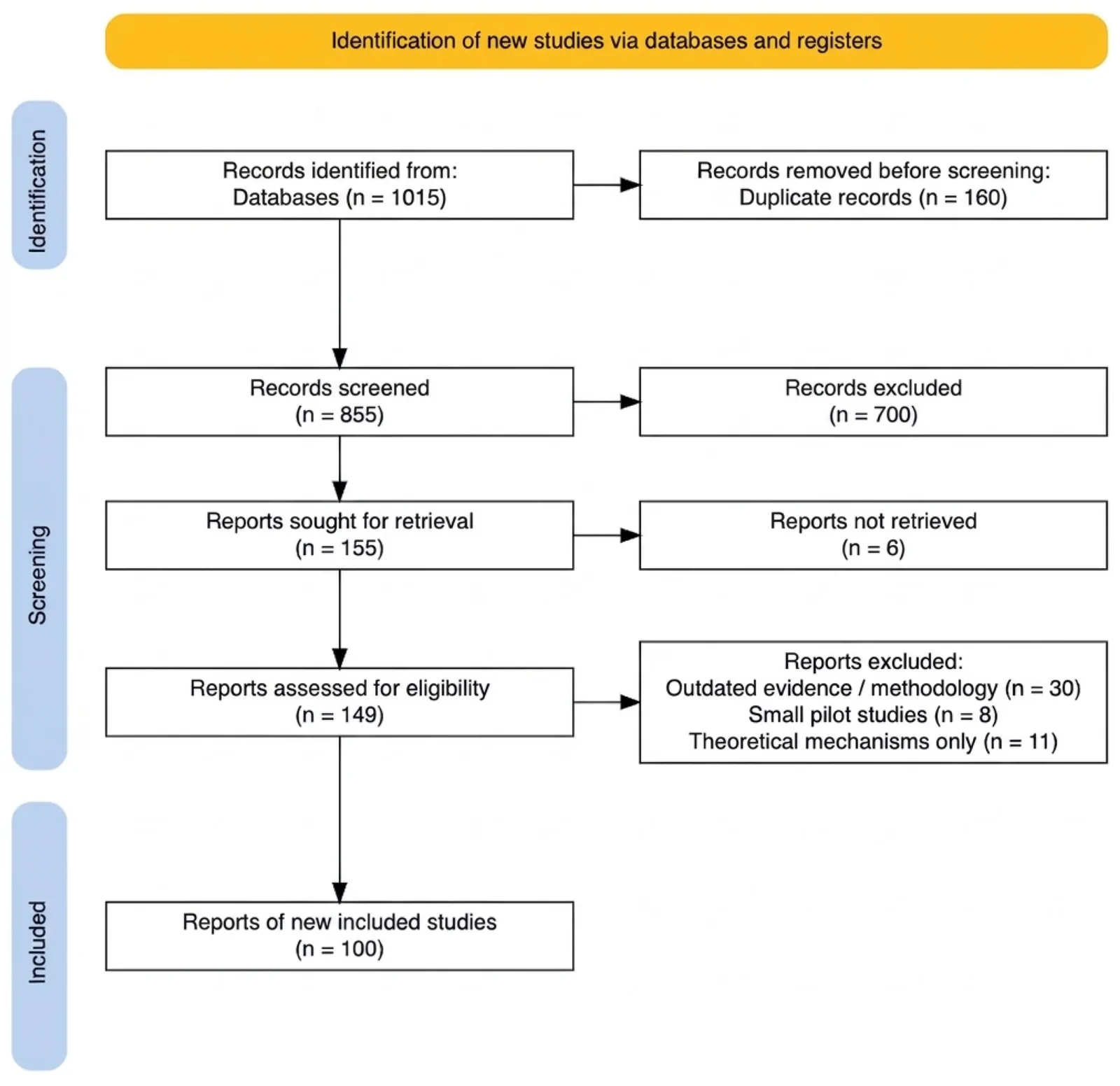
PRISMA flow diagram illustrating the literature search strategy, screening process, and exclusion criteria applied to select the final 100 studies included in this review.

The initial search yielded a total of 1015 records. After the removal of 160 duplicate records, 855 titles and abstracts were screened. Of these, 700 records were excluded due to being off-topic, being reviews of already included primary literature, involving incorrect patient populations, or being published in non-English languages, and 6 reports could not be retrieved. The remaining 149 reports were assessed for full-text eligibility. A further 49 reports were excluded due to outdated methodology, being limited to small pilot studies, or describing theoretical mechanisms lacking solid preclinical or clinical validation. Ultimately, 100 studies met the inclusion criteria and were incorporated into this review (Figure 1).

## 2. Relevant Sections

### 2.1. The Pathophysiological Continuum: From Dystrophin Deficiency to Myocardial Fibrosis

The pathogenesis of DMD/BMD cardiomyopathy differs in several important respects from that of conventional hemodynamic or ischemic cardiomyopathies. The primary molecular defect—an absence or severe truncation of the cytoskeletal protein dystrophin—impairs the structural integrity of the cardiomyocyte sarcolemma and increases its susceptibility to mechanical stress. Repetitive membrane injury may promote abnormal calcium influx and downstream activation of cellular pathways leading to cardiomyocyte injury, inflammation, and progressive replacement of myocardial tissue by fibrotic and fatty tissue [2,4].

Unlike idiopathic dilated cardiomyopathy, where ventricular chamber remodeling and systolic dysfunction may develop relatively early in the clinical course, dystrophin-associated cardiomyopathy is characterized by a prolonged subclinical phase in which myocardial injury and fibrosis may accumulate despite preserved global ventricular systolic function [4]. Fibrotic involvement shows a preferential regional distribution, with early abnormalities frequently involving the posterolateral and inferolateral left ventricular myocardium, and may subsequently extend to other myocardial segments as disease progresses [5]. Importantly, the extent and timing of cardiac involvement do not necessarily parallel the severity of skeletal muscle disease; in BMD in particular, clinically significant cardiomyopathy may develop despite relatively preserved skeletal muscle function and ambulation [6].

Furthermore, the dystrophic heart does not operate in isolation. Progressive respiratory muscle weakness, thoracic deformity, altered ventricular loading conditions, and persistent sinus tachycardia may interact with the intrinsic myocardial substrate to further increase cardiovascular vulnerability, particularly in advanced disease [2,5]. These observations help explain why conventional global measures such as LVEF may remain preserved despite substantial regional myocardial injury, fibrosis, and mechanical dysfunction. Rather than replacing LVEF, advanced imaging modalities therefore provide complementary information on the timing, distribution, and progression of myocardial disease, supporting a shift toward earlier detection and potentially earlier cardioprotective intervention.

### 2.2. Early Subclinical Detection: The Prognostic Role of Advanced Imaging

#### 2.2.1. The Tissue Doppler Imaging

The traditional reliance on LVEF systematically underestimates the onset and severity of early dystrophic cardiomyopathy. Tissue Doppler Imaging (TDI) provided some of the earliest evidence that subtle myocardial abnormalities may precede overt systolic dysfunction, with studies demonstrating alterations in diastolic indices, including annular velocities and the Tei index, before clinically apparent ventricular dysfunction develops [7]. Pioneering myocardial deformation studies further demonstrated that regional strain abnormalities may be detectable despite preserved global systolic function, particularly in the left ventricular posterior wall. In these studies, radial strain was reduced in specific myocardial regions, with abnormal deformation patterns involving the outer myocardial layers that appeared to parallel the characteristic regional distribution of early fibrotic replacement [8]. Building on these observations, transmural myocardial strain analysis identified an abnormal “notched” contraction pattern in the posterior wall, which was associated with the subsequent development of regional wall-motion abnormalities within the following year [9]. Although these findings established the potential of deformation imaging for early phenotyping, their relatively small study populations and the technical limitations of early TDI-based approaches limit their use as stand-alone clinical decision-making tools. Nevertheless, they provided an important foundation for the subsequent adoption of speckle-tracking echocardiography and CMR-based tissue characterization in dystrophin-associated cardiomyopathy.

#### 2.2.2. Two-Dimensional Speckle-Tracking Echocardiography

Two-dimensional speckle-tracking echocardiography (2D-STE) overcomes the inherent angle dependence of Tissue Doppler Imaging, establishing Global Longitudinal Strain (GLS) as a sensitive marker of early myocardial dysfunction. In DMD cohorts, GLS impairment localizes very early to the basal inferolateral and inferior walls [7]. This regional pattern of myocardial deformation may be present despite preserved LVEF and has been associated with early ventricular mechanical abnormalities [10]. Specifically, 2D-STE reveals pronounced opposing-wall delays between the inferoseptal and anterolateral segments, indicating that mechanical dyssynchrony may precede overt global systolic dysfunction and can occur in the absence of electrical dyssynchrony [10]. These findings support the use of GLS and regional strain analysis as complementary markers of early cardiac involvement, while their role as independent treatment thresholds remains to be established. Advanced layer-specific tracking further deconstructs this progression, revealing a complex biomechanical paradox. While traditional models and radial TDI suggest an epicardial-first progression, layer-specific 2D-STE reveals that circumferential strain deterioration actually begins in the middle myocardial layer and progresses to the endocardium, while the epicardial layer may paradoxically remain relatively preserved or deteriorate later [11]. These apparently discordant findings indicate that structural fibrosis and functional myocardial deformation may evolve through partially overlapping but not necessarily synchronous processes.

#### 2.2.3. Disease Trajectory and Biochemical Correlates

The prognostic superiority of GLS is particularly evident in longitudinal tracking across the dystrophic continuum. In DMD boys, the physiological decline of global left ventricular (LV) longitudinal strain with age is dramatically accelerated, worsening by an additional 0.34% per year compared to healthy controls [12]. Similarly, in BMD cohorts, patients exhibit a steady, significant annual deterioration in LV GLS (approximately 0.60%) over a 2-year period, while LVEF and LV volumes remain deceptively stable [13]. Furthermore, this declining GLS correlates significantly with elevations in cardiac biomarkers, including high-sensitivity Troponin I and N-terminal pro-brain natriuretic peptide (NT-proBNP), supporting an association between worsening myocardial deformation and biochemical evidence of myocardial injury and neurohormonal stress [13,14].

#### 2.2.4. 3D Echocardiography

While 2D-STE is invaluable, it remains limited by out-of-plane speckle motion. The evolution toward three-dimensional (3D) STE allows for a simultaneous, comprehensive assessment of ventricular mechanics from a single volumetric dataset [15]. In pediatric DMD populations, 3D Global Area Strain (GAS)—which integrates both longitudinal and circumferential shortening—has shown good diagnostic performance (AUC 0.80) for the detection of subclinical LV dysfunction [15]. Furthermore, 3D tracking confirms an age-related decline in myocardial function, supporting its potential role in the assessment of cardiomyopathy severity even when LVEF remains preserved.

#### 2.2.5. CMR Functional Tracking and Prognosis

Functional deformation assessment can be further refined by Cardiovascular Magnetic Resonance (CMR). While CMR is widely recognized as the reference technique for detecting structural fibrosis through late gadolinium enhancement (LGE), tagged CMR assessment of circumferential strain has demonstrated progressive deterioration of myocardial mechanics over time, even in patients whose LVEF remains stable or appears to improve [16]. Recent studies using CMR tissue tracking further emphasize this temporal gap, with regional strain abnormalities detectable as early as 7–10 years of age in boys with DMD, preceding both overt LVEF decline and, in some patients, LGE positivity [17]. Reduced global radial and circumferential strain have been shown to predict the subsequent development of DMD-associated cardiomyopathy (LVEF < 50%) up to 32 months before overt systolic dysfunction. More recently, 4D CMR kinematic analysis has demonstrated that regional strain metrics, particularly basal circumferential and surface area strain, may provide prognostic information beyond conventional functional parameters [18,19]. Geometry-independent 3D minimum principal strain has also emerged as a promising prognostic marker, with higher-risk patients showing an approximately 5.5-fold increased risk of major adverse cardiac events compared with those without these abnormalities [20,21].

#### 2.2.6. Parametric Mapping: Tissue Characterization Before Scarring

Parametric CMR mapping provides quantitative assessment of diffuse myocardial remodeling and may detect abnormalities before overt focal scar becomes apparent on LGE. Patients with DMD exhibit elevated native T1 values and increased extracellular volume (ECV), including in the presence of preserved LVEF and absent LGE [22,23,24]. Layer-specific analyses suggest a predominantly epicardial distribution of early native T1 abnormalities, while regional differences in native T1 and ECV, particularly between the relatively preserved interventricular septum and the more affected left ventricular posterior wall, have been associated with subsequent changes in LVEF. However, in advanced disease characterized by substantial fibrofatty replacement, clinicians should be aware of the “T1 paradox,” whereby the increased fat content may contribute to lower measured myocardial T1 values despite more advanced myocardial disease [25,26].

#### 2.2.7. Beyond the Left Ventricle: Biventricular and Atrial Remodeling

Myocardial involvement in dystrophinopathies is not confined to the left ventricle. Pre-contrast T1 mapping of the right ventricle (RV) reveals elevated values that negatively correlate with RV ejection fraction, suggesting concomitant right ventricular microstructural involvement that may become particularly relevant as respiratory insufficiency and RV afterload increase [27]. Additionally, left atrial (LA) phasic function—specifically reservoir and conduit strain—decreases significantly as the disease progresses. This impairment in LA compliance may represent an early marker of diastolic dysfunction and impaired ventricular relaxation, even in the absence of LV dilation [28].

#### 2.2.8. LGE and Geometric Remodeling

Focal replacement fibrosis, visualized by LGE, is an important marker of disease severity and adverse cardiac prognosis. The age at which LGE first appears may influence disease trajectory; patients developing “early LGE” before 14 years of age experience a significantly accelerated rate of systolic decline [29]. Longitudinally, the prevalence of a transmural LGE pattern nearly doubles over a 4-year period [30]. The extent and distribution of LGE are associated with heart failure and ventricular arrhythmias, providing prognostic information even in patients with relatively preserved LVEF, while advanced LGE grades have also been associated with QT dispersion and mortality [31]. Finally, integrating cardiac geometry with tissue characterization may further refine risk stratification: the development of LV concentric hypertrophy on CMR has been identified as an independent predictor of composite cardiac events, providing incremental prognostic information beyond age and LGE positivity [32,33].

### 2.3. Prophylactic Interventions and Their Limits in the Pediatric Population

Historically, corticosteroid therapy has been the cornerstone of DMD management to prolong ambulation, with early echocardiographic studies suggesting a concurrent delay in the onset of ventricular dysfunction [34]. However, the traditional reliance on load-dependent, late-stage markers such as fractional shortening and LVEF may underestimate the effects of corticosteroids on the evolving dystrophic myocardium.

Advanced CMR provides a more nuanced, and sometimes contradictory, perspective on the “steroid paradox.” On the one hand, long-term deflazacort therapy has been associated with higher myocardial T2 relaxation times and better LVEF preservation in asymptomatic patients compared with untreated boys [35]. Because myocardial T2 relaxation times in DMD progressively decrease below normal values as a consequence of altered tissue water–fat composition and fibrous replacement, the preservation of higher T2 values in deflazacort-treated patients reflects better preservation of normal myocardial architecture and less progressive fibrosis, rather than reduced myocardial edema [35]. Consistently, large longitudinal CMR data have shown that longer corticosteroid treatment duration (predominantly deflazacort) is associated with an attenuated age-related increase in LGE-defined myocardial fibrosis burden, although this protective effect remains modest and LVEF decline is ultimately driven by the number of fibrotic segments [36].

Despite these structural associations, functional stabilization remains elusive. CMR tracking of circumferential strain indicates that mechanical deterioration may continue even in boys receiving combined therapy with corticosteroids and ACEi or angiotensin receptor blockers (ARBs) [37]. This continued subclinical decline raises questions regarding the cardiovascular effects of prolonged steroid exposure, including potential contributions from fluid retention and hypertension. Supporting the complexity of this issue, preclinical murine studies have shown that prednisolone may exacerbate histological evidence of myocardial injury (assessed by the percentage of tissue area demonstrating IgG infiltration and collagen staining) and attenuate the functional benefits of prophylactic ACEi and mineralocorticoid receptor antagonists (MRAs). Thus, the cardioprotective effects of corticosteroids remain biologically complex and may differ between structural remodeling and functional myocardial progression [38].

Moving beyond symptom management, dystrophin-restoring strategies such as exon skipping are emerging as potential disease-modifying approaches. Systemic peptide-conjugated phosphorodiamidate morpholino oligomers have restored cardiac dystrophin and improved early right and left ventricular dysfunction in dystrophic murine models. In vitro studies using human induced pluripotent stem cell-derived cardiomyocytes have similarly demonstrated that antisense oligonucleotide-mediated exon skipping and minigene transfer can restore dystrophin expression to functionally relevant levels (approximately 30% of wild-type). Nevertheless, whether these approaches can prevent or substantially delay myocardial fibroadipose replacement in humans remains uncertain, underscoring the continued importance of established pharmacological cardioprotection [39,40].

The limitations of a traditional “watch and wait” approach are illustrated by rescue-therapy data. When ACE inhibitors or beta-blockers are initiated only after overt LV systolic dysfunction develops, therapeutic response may be variable, with some patients showing continued ventricular dilation despite treatment [41,42]. In BMD, earlier ACE inhibitor treatment, including at LVEF thresholds above those traditionally used for overt systolic dysfunction, has been associated with improved cardiac outcomes, although the available evidence remains largely observational [43,44]. Thus, these data support consideration of earlier intervention while highlighting the need for prospective validation.

For DMD, seminal trials of prophylactic perindopril initiated before the onset of overt LV dysfunction demonstrated a delayed development of systolic dysfunction [45,46,47]. Registry data have subsequently supported an association between prophylactic ACE inhibitor use and improved overall survival, strengthening the rationale for early renin–angiotensin system inhibition. CMR studies have further suggested that early treatment with perindopril in combination with corticosteroids may be associated with a lower burden of myocardial fibrosis, although the specific contribution of each treatment remains difficult to disentangle [46].

The evidence for mineralocorticoid receptor antagonists further supports an imaging-guided approach. In boys with early DMD cardiomyopathy and preserved LVEF, the addition of eplerenone to background ACE inhibitor or ARB therapy attenuated the decline in LV circumferential strain [48]. The multicenter AIDMD trial subsequently demonstrated that spironolactone was noninferior to eplerenone for the preservation of cardiac contractility over 12 months, supporting spironolactone as a practical alternative when MRA therapy is indicated [49].

The role of beta-blockers in early DMD cardiomyopathy remains less clearly defined. Retrospective studies suggest potential benefit, whereas randomized trials have not consistently demonstrated a significant effect on conventional echocardiographic endpoints such as fractional shortening [44,50]. This may partly reflect the limitations of load-dependent endpoints in early dystrophic cardiomyopathy, but current evidence remains insufficient to establish a universal prophylactic strategy for beta-blockers before overt ventricular dysfunction. Ongoing studies, including the DMD Heart Protection Study, may help clarify the role of combined ACE inhibitor and beta-blocker therapy in patients with preserved conventional measures of cardiac function [50,51,52,53].

### 2.4. Navigating the Transitional Age: Integrating Adult GDMT

As advances in respiratory care extend survival in patients with DMD into young adulthood, an increasing number of patients are transitioning from pediatric networks to adult cardiology clinics. This transition exposes an important methodological gap: adult heart failure guidelines rely heavily on conventional LVEF-based definitions of systolic dysfunction, whereas dystrophin-associated cardiomyopathy may show substantial myocardial disease before or in excess of the abnormalities captured by LVEF alone. Consequently, direct extrapolation of adult heart failure strategies requires consideration of the distinct pathophysiology, hemodynamic profile, and comorbidities of patients with dystrophinopathy [54,55]. The integration of angiotensin receptor–neprilysin inhibitors (ARNI), a Class I recommendation for adult heart failure with reduced ejection fraction, illustrates the limited evidence available in DMD. Small, non-randomized cohorts of symptomatic DMD patients treated with sacubitril/valsartan have reported modest LVEF improvements (e.g., from 31% to 38%) and clinical stabilization over approximately 7 months [56,57]. However, hypotension may limit treatment in selected patients with advanced dystrophin-associated cardiomyopathy: forcing vasodilation in a fragile cardiovascular system is perilous. DMD patients typically exhibit constitutionally low baseline blood pressure and minimal cardiac reserve. Standard adult-style ARNI administration in this population can provoke recurrent, severe hypotension, reflex atrial tachycardia, and cardiogenic shock, supporting cautious dose initiation and titration rather than direct application of conventional adult dosing strategies.

The ubiquitous presence of resting sinus tachycardia in DMD presents another therapeutic consideration. While heart-rate reduction is a standard component of heart failure management, concerns have been raised regarding the potential impact of excessive chronotropic reduction in patients with limited cardiac reserve [58]. Preliminary clinical data suggest that ivabradine may reduce heart rate while maintaining tolerability in DMD patients, with reported improvements in LVEF in small observational cohorts [59]. Mechanistic studies in DMD models further suggest potential effects on intracellular calcium handling and myocardial function [60,61]; however, these findings remain hypothesis-generating and require clinical validation.

Sodium-glucose cotransporter-2 inhibitors (SGLT2i) represent an emerging therapeutic strategy with both clinical and mechanistic interest in dystrophin-associated cardiomyopathy. Preclinical studies suggest potential effects on sodium-channel function, myocardial metabolism, and fibrosis [62,63,64]. More recently, real-world ACTION registry data have provided initial evidence of feasibility and tolerability in patients with DMD, showing stabilized LVEF with a low (18%) incidence of adverse events and minimal risk of acute kidney injury (5%) or urinary tract infections (2.5%) [65,66]. However, the absence of a control group limits conclusions regarding efficacy.

However, the metabolic benefits of SGLT2i come with a strict DMD-specific caveat. Because patients with advanced DMD possess severely diminished skeletal muscle mass, their glycogen reserves are inherently depleted. This altered physiology renders them highly susceptible to euglycemic ketoacidosis. Episodes of dysphagia, delayed intestinal motility, or fasting during acute illness can rapidly precipitate euglycemic ketoacidosis without significant hyperglycemia, requiring immediate drug cessation and dextrose infusion [67].

Recognizing these unique physiological boundaries, ongoing research aims to standardize SGLT2i use in this vulnerable population. Protocols such as the REDMED trial (Repurposing Empagliflozin for DMD-Associated Cardiomyopathy) are currently underway to establish precise pharmacokinetic profiles and define optimal pediatric dosing regimens, ensuring that future interventions are both safe and targeted [68]. Ultimately, integrating adult GDMT during the transitional period requires an individualized approach that balances the potential benefits of contemporary heart failure therapies against the hemodynamic, metabolic, and respiratory characteristics of dystrophin-associated disease. Table 1 summarizes the most widely used pharmaceutical agents.

### 2.5. Advanced Device Therapies: Efficacy and Competing Risks

#### 2.5.1. Arrhythmias and the Limits of Ambulatory Monitoring

Clinically significant arrhythmias are uncommon in DMD patients with preserved cardiac function. Ambulatory monitoring has shown that complex ventricular arrhythmias, including non-sustained ventricular tachycardia, become more frequent with advanced LV dysfunction, particularly at LVEF values below 35% [69]. However, long-term monitoring studies suggest that arrhythmia burden may correlate more closely with left ventricular volume and the extent of myocardial fibrosis, assessed by extracellular volume and LGE, than with LVEF alone [70]. Thus, ventricular arrhythmias appear to reflect both the severity of ventricular dysfunction and the underlying myocardial substrate, rather than representing an isolated electrical phenotype.

#### 2.5.2. ICD Implantation

The prevention of sudden cardiac death (SCD) using implantable cardioverter-defibrillators (ICDs) remains highly controversial in dystrophinopathies. Standard primary-prevention criteria derived from non-dystrophic heart failure populations, including an LVEF threshold of <35%, may not fully capture arrhythmic risk in DMD, as myocardial fibrosis and other features of the dystrophic substrate may contribute to ventricular arrhythmogenesis independently of LVEF [71]. A multidimensional approach incorporating LGE, speckle-tracking echocardiography, and other clinical and electrophysiological parameters has therefore been proposed to refine risk stratification.

When considering device therapy, clinicians must weigh potential arrhythmic benefit against substantial competing risks. Advanced DMD is frequently associated with severe restrictive respiratory disease and kyphoscoliosis, which may increase perioperative morbidity. Device implantation in tracheotomized or severely disabled patients has been associated with non-negligible procedural complications, including pneumothorax, while inappropriate shocks may also occur in this population [71,72,73]. Despite these challenges, qualitative studies indicate that patients and caregivers value individualized and transparent discussions regarding SCD prevention and device therapy. ICD decisions should therefore be individualized, taking into account myocardial substrate, ventricular function, arrhythmic history, respiratory status, life expectancy, and patient preferences.

#### 2.5.3. Cardiac Resynchronization Therapy

The application of cardiac resynchronization therapy (CRT) highlights an important distinction between mechanical and electrical dyssynchrony in DMD. CMR studies have demonstrated mechanical dyssynchrony in a subset of patients with advanced cardiac involvement and myocardial fibrosis, sometimes despite a normal QRS duration [74]. Because isolated mechanical dyssynchrony does not fulfill conventional electrical criteria for CRT, its therapeutic significance remains uncertain and evidence supporting CRT in this setting is lacking.

For the minority of dystrophinopathic patients who develop established electrical dyssynchrony, such as left bundle branch block with a markedly prolonged QRS complex, CRT may be considered according to standard heart failure criteria. Published evidence in DMD and BMD is currently limited largely to individual cases and small clinical experiences, although sustained improvements in ventricular function and heart failure status have been reported [75,76].

#### 2.5.4. Left Ventricular Assist Devices and Heart Transplantation

Heart transplantation presents substantial differences across muscular dystrophies. In BMD and selected forms of limb-girdle and Emery–Dreifuss muscular dystrophy with advanced cardiomyopathy but sufficient extracardiac reserve, heart transplantation and continuous-flow left ventricular assist devices (cf-LVADs) may represent feasible advanced heart failure options. Registry and case-series data suggest that post-transplant outcomes can be comparable to those observed in other non-ischemic cardiomyopathies [77,78]. In selected patients, restoration of cardiac output may also improve functional capacity and, in some cases, partially reverse severe deconditioning [79]. In DMD, by contrast, severe respiratory muscle weakness, thoracic deformity, skeletal muscle disease, and multisystem involvement substantially limit candidacy for transplantation, which therefore remains restricted to highly selected patients.

Consequently, LVAD support in DMD has most often been considered as Destination Therapy rather than as a bridge to transplantation [77,78,79]. Implantation in patients with severe kyphoscoliosis and restrictive respiratory disease is technically challenging, and surgical strategies must minimize disruption of diaphragmatic and respiratory mechanics. Experience with less invasive and off-pump implantation techniques has demonstrated technical feasibility in selected patients, although postoperative right ventricular failure, bleeding, and respiratory complications remain important concerns [80,81].

While LVAD support can improve hemodynamics and reverse ventricular unloading-related remodeling, the underlying dystrophinopathy continues to affect the myocardium and skeletal muscle. Persistent myocardial atrophy despite LV reverse remodeling has been reported during mechanical support [82]. Thus, LVAD therapy should be viewed as an advanced supportive strategy rather than a treatment of the underlying disease, with careful multidisciplinary assessment and shared decision-making regarding expected benefit, treatment burden, and goals of care.

## 3. Discussion

Despite clear pathophysiological evidence and unprecedented imaging advancements, a significant translational gap remains in the real-world management of dystrophinopathy. Recent data from the HOPE-MD registry reveal that only 16% of young adults with DMD/BMD and heart failure with reduced ejection fraction receive optimal guideline-directed medical therapy [83,84]. While traditional agents like ACE inhibitors are frequently prescribed, cornerstone anti-remodeling therapies are noticeably underutilized: 51% of eligible patients lack MRAs, and 78% are missing SGLT2 inhibitors [84]. The North American ACTION registry reflects a similarly challenging landscape: while ACE inhibitor use exceeds 91%, beta-blocker utilization stalls at 52.8%, and SGLT2i adoption is a mere 7.6% [84,85].

These findings suggest that the challenge is no longer limited to recognizing cardiac involvement, but also to translating existing and emerging evidence into consistent clinical practice.

While advanced imaging (including strain, parametric mapping, and LGE) consistently detects myocardial injury years before LVEF declines, diagnostic sensitivity has outpaced therapeutic validation. A critical distinction must be maintained between an imaging biomarker’s prognostic value and proof that biomarker-guided therapy improves clinical outcomes. Future studies should therefore move beyond demonstrating that a biomarker predicts LVEF decline and establish whether biomarker-guided intervention can alter the natural history of dystrophin-associated cardiomyopathy.

At present, the strongest evidence for preventive therapy remains centered on early renin–angiotensin system inhibition and mineralocorticoid receptor antagonism, whereas the clinical role of newer therapies remains less well established [20,22,86,87,88,89,90].

Bridging this evidence-to-practice gap is particularly critical during the transition from pediatric to adult care. Rather than mechanically replacing pediatric prophylactic regimens with standard adult LVEF-driven protocols, clinicians must integrate contemporary pharmacological options (ARNI, SGLT2i, ivabradine) [59,65,66,67,68,91,92,93] and advanced device or surgical strategies (ICD, CRT, LVAD, transplantation) [71,77,78,79,80,81,82,83] into an individualized framework that accounts for the unique hemodynamic fragility, metabolic vulnerability, myocardial fibrosis burden, and respiratory competing risks of dystrophinopathy. Multidisciplinary assessment and shared decision-making are particularly important when considering invasive therapies in advanced DMD.

Collaborative networks such as ACTION may facilitate harmonization of surveillance, treatment strategies, and advanced heart failure referral across institutions [85]. A longitudinal, multidisciplinary model integrating neuromuscular, pediatric and adult cardiology, advanced imaging, respiratory medicine, and palliative care may ultimately provide the most coherent framework for patients with progressive dystrophin-associated cardiomyopathy.

## 4. Conclusions

Cardiovascular management of DMD/BMD is increasingly moving from a predominantly LVEF-driven approach toward earlier recognition of myocardial disease using strain imaging and CMR. Early initiation of established cardioprotective therapies remains the cornerstone of management, while contemporary heart failure therapies and device-based interventions are gradually being integrated into selected patients with more advanced disease. The major unmet need is to determine whether the increasingly sensitive detection of subclinical myocardial abnormalities can be translated into earlier, biomarker-guided interventions that ultimately improve clinically meaningful outcomes. A coordinated multidisciplinary approach spanning pediatric and adult care will be essential to address this challenge across the course of dystrophin-associated cardiomyopathy.

## 5. Future Directions

While current guideline-directed therapies primarily target the downstream consequences of dystrophin deficiency, emerging approaches aim to interfere more directly with the molecular and cellular mechanisms underlying myocardial injury. Experimental strategies targeting calcium handling, oxidative stress, ion-channel dysfunction, and myocardial remodeling are therefore of considerable interest. Specifically, mechanical stress in dystrophin-deficient cardiomyocytes promotes the abnormal sarcolemmal translocation of the stretch-sensitive channel TRPV2, driving excessive calcium influx, protease activation, and profibrotic signaling. Clinical experience with the TRPV2 inhibitor tranilast suggests that blocking sarcolemmal TRPV2 accumulation may help stabilize natriuretic peptides and cardiac function in advanced dystrophic heart failure, although the available evidence remains limited and requires confirmation in controlled studies [94]. Similarly, preliminary clinical and preclinical findings with tamoxifen and simvastatin warrant further investigation but should currently be regarded as experimental rather than established cardioprotective therapies [95,96].

The translational gap between preclinical efficacy and clinical benefit remains a major challenge in dystrophin-associated cardiomyopathy. The divergent results obtained with tadalafil—showing benefit in dystrophic animal models but no clear clinical benefit in DMD—illustrate the limitations of extrapolating mechanistic or preclinical findings directly to patients [97,98]. Regenerative approaches such as mesoangioblast-based therapy further suggest that the timing of intervention relative to myocardial fibrosis may be critical, as efficacy appears to decline once extensive structural damage is established [99].

Parallel preclinical efforts target electrophysiological and redox pathways that perpetuate myocardial dysfunction. Because dystrophin and utrophin regulate the cardiac voltage-gated sodium channel *Na*_V_1.5 via syntrophin adaptor proteins, loss of sarcolemmal anchoring reduces *Na*_V_1.5 expression and peak sodium currents, slowing action potential upstroke velocity and causing conduction abnormalities that may be mitigated by utrophin preservation or SGLT2 inhibition [62,63,97]. Concurrently, upregulation of NADPH oxidase 4 (NOX4) in dystrophic cardiomyocytes amplifies reactive oxygen species production, mitochondrial bioenergetic failure, and myofibroblast-driven fibrosis—a cascade attenuated in dystrophic murine models by the NOX1/4 inhibitor setanaxib [100].

Future studies should therefore move beyond the identification of novel targets and focus on biomarker-defined therapeutic windows, clinically meaningful endpoints, and prospective evaluation of combination strategies. Advanced CMR and myocardial strain may help identify patients at earlier stages of myocardial disease, but their ultimate value will depend on demonstrating that imaging-guided intervention alters the subsequent trajectory of cardiomyopathy.

### Call to Action

The rapidly evolving landscape of DMD/BMD, reshaped by life-prolonging respiratory interventions (e.g., NIV, CPAP) and novel disease-modifying treatments (such as exon-skipping and givinostat), renders the classical heart failure paradigm increasingly inadequate. Translating evidence from non-dystrophic adult populations to this fragile cohort carries inherent physiological risks that cannot be responsibly mitigated by expert consensus alone. Therefore, this review highlights the urgent need for dedicated, multi-center registries and specifically designed clinical trials. To be clinically meaningful, future research must move beyond conventional load-dependent endpoints like LVEF, embracing multiparametric imaging (such as GLS and LGE) as primary surrogates of disease progression. Crucially, trial designs must systematically account for the unique confounding factors of this population, particularly the hemodynamic impact of chronic positive-pressure ventilation and the potential cardiovascular effects of emerging genetic therapies. A rigorous, phenotype-driven research agenda is essential to safely and effectively guide cardiovascular management in this unprecedented transitional generation of patients.

## Figures and Tables

**Table 1:** Summary of pharmacological cardioprotective strategies across the dystrophinopathy continuum, categorized by clinical stage, therapeutic class, typical administration window, and current level of clinical evidence.

Current Level of Evidence	Typical Clinical Window	Therapeutic Agents	Disease Stage
Observational cohorts and longitudinal CMR studies	Pediatric prophylactic initiation	Corticosteroids *(Deflazacort, Prednisone)*	Pre-symptomatic/Early Subclinical Stage
Established	Pediatric prophylactic initiation (before overt LVEF decline)	ACE Inhibitors/ARBs *(Perindopril, Enalapril, Losartan)*	Pre-symptomatic/Early Subclinical Stage
Established	Childhood/Adolescence	MRAs *(Eplerenone, Spironolactone)*	Early Myocardial Injury/Fibrotic Remodeling
Mixed	Variable	Beta-Blockers *(Carvedilol, Metoprolol)*	Early to Overt Systolic Dysfunction/Sinus Tachycardia
Emerging/Limited	Adolescents/young adults with symptomatic HFrEF or LVEF decline	ARNI *(Sacubitril/Valsartan)*	Transitional Age and Overt Systolic Dysfunction
Emerging/Limited	Adolescents/young adults with symptomatic HFrEF or LVEF decline	SGLT2 Inhibitors *(Dapagliflozin, Empagliflozin)*	Transitional Age and Overt Systolic Dysfunction
Preliminary	Transitional age and advanced/end-stage cardiomyopathy	I_f_ Channel Inhibitor *(Ivabradine)*	Refractory Sinus Tachycardia

## Data Availability

No new data were created or analyzed in this study. Data sharing is not applicable to this article.

## Abbreviations

The following abbreviations are used in this manuscript:

DMD	Duchenne muscular dystrophy
BMD	Becker muscular dystrophy
GLS	Global Longitudinal Strain
ACE	Angiotensin-converting-enzyme
GMDT	Guideline-directed medical therapies
SGLT2	Sodium-Glucose Co-Transporter 2
ICD	Implantable cardioverter-defibrillator
CRT	Cardiac Resynchronization Therapy
LVEF	Left ventricular ejection fraction
ARNI	Angiotensin Receptor-Neprilysin Inhibitors
CMR	Cardiovascular Magnetic Resonance
TDI	Tissue Doppler Imaging
STE	Speckle-tracking echocardiography
NT-proBNP	N-terminal pro-brain natriuretic peptide
ECV	Extracellular volume
LGE	Late gadolinium enhancement
RV	Right ventricle
LA	Left atrium
LV	Left ventricle
ARB	Angiotensin receptor blockers
MRA	Mineralocorticoid receptor antagonists
SCD	Sudden cardiac death
LVAD	Left ventricular assist devices
